# Management of vascular risk in people with multiple sclerosis at the time of diagnosis in England: A population-based study

**DOI:** 10.1177/13524585231164296

**Published:** 2023-04-07

**Authors:** Raffaele Palladino, Ruth Ann Marrie, Azeem Majeed, Jeremy Chataway

**Affiliations:** Department of Primary Care and Public Health, School of Public Health, Imperial College of London, London, UK/Department of Public Health, Federico II University, Naples, Italy; Departments of Medicine and Community Health Sciences, Max Rady College of Medicine, Rady Faculty of Health Sciences, University of Manitoba, Winnipeg, MB, Canada; Department of Primary Care and Public Health, School of Public Health, Imperial College of London, London, UK; Queen Square Multiple Sclerosis Centre, Department of Neuroinflammation, UCL Queen Square Institute of Neurology, Faculty of Brain Sciences, University College London, London, UK/National Institute for Health Research, University College London Hospitals, Biomedical Research Centre, London, UK

**Keywords:** Multiple sclerosis, epidemiology, vascular management, diabetes, hypertension, BMI

## Abstract

**Background:**

Vascular management in People with Multiple Sclerosis (PwMS) is important given the higher vascular burden than the general population, associated with increased disability and mortality.

**Objectives:**

We assessed differences in the prevalence of type 2 diabetes and hypertension; and the use of antidiabetic, antihypertensive and lipid-lowering medications at the time of the MS diagnosis.

**Methods:**

This is a population-based study including PwMS and matched controls between 1987 and 2018 in England.

**Results:**

We identified 12,251 PwMS and 72,572 matched controls. PwMS had a 30% increased prevalence of type 2 diabetes (95% confidence interval (CI) = 1.19, 1.42). Among those with type 2 diabetes, PwMS had a 56% lower prevalence of antidiabetic usage (95% CI = 0.33, 0.58). Prevalence of hypertension was 6% greater in PwMS (95% CI = 1.05, 1.06), but in those with hypertension, usage of antihypertensive was 66% lower in PwMS (95% CI = 0.28, 0.42) than controls. Treatment with lipid-lowering medications was 63% lower in PwMS (95% CI = 0.54, 0.74). PwMS had a 0.4-mm Hg lower systolic blood pressure (95% CI = −0.60, −0.13). 3.8% of PwMS were frail.

**Conclusion:**

At the time of diagnosis, PwMS have an increased prevalence of vascular risk factors, including hypertension and diabetes though paradoxically, there is poorer treatment. Clinical guidelines supporting appropriate vascular assessment and management in PwMS should be developed.

## Introduction

People with Multiple Sclerosis (PwMS) have an increased incidence and prevalence of vascular comorbidities, including diabetes, hypertension and dyslipidemia, as compared with people without MS.^1–7^ This contributes to the 30% greater risk of macrovascular disease.^1,4^ Moreover, as the vascular burden rises over time,^2^ it is associated with increased disability worsening,^8^ healthcare utilization,^9^ and all-cause/vascular mortality.^1,3^

Given this background, the assessment and management of vascular comorbidities is vital, particularly in the very early stages. However, there is a paucity of studies examining vascular risk factor control in PwMS. A Canadian retrospective cohort study of 971,799 individuals identified using a primary care database between 2014 and 2016, of whom 2926 were PwMS, concluded that MS was not associated with poorer control of blood pressure and diabetes or difference in the median number of medications used to treat these conditions.^4^ Furthermore, a previous study conducted in Italy reported higher use of antihypertensive in PwMS than matched controls.^10^ However, the findings from these studies may not apply to other health systems with differing access to and systems of care and with different treatment guidelines.^11^

To take this forward, we used a large data set representative of the English population to assess the (1) the prevalence of vascular risk factors; (2) and the intensity of management of vascular risk in PwMS, at the *time of diagnosis* as compared with a matched control population. A novel aspect was the incorporation of the validated electronic frailty index (eFI) as a proxy of MS disability, as previous research has shown that frailty indices are strongly associated with MS disease duration, disability and fatigue.^12^

## Methods

### Study design

We conducted a population-based cross-sectional study which included PwMS and matched controls registered with general practices in England, diagnosed between 1 January 1987 and 30 September 2018. The Independent Scientific Advisory Committee of the CPRD (protocol no. 18_279R) granted ethics approval.

### Data source

Data were drawn from the UK Clinical Practice Research Datalink (CPRD) GOLD, one of the largest databases of electronic medical records globally.^13^ The CPRD GOLD holds anonymized routinely collected longitudinal primary care records from general practices using the same software system (Vision^®^) who have agreed at practice level to provide data monthly.^13^ The database includes information on all patients registered with the participating practices unless they have individually requested to opt out of data sharing. The database covers approximately 7% of the UK population; it is representative with respect to age, sex and ethnicity.^14^ As linkage to Hospital Episode Statistics and Office for National Statistics mortality data is available only for the English data set,^13^ we limited the study to individuals registered with English general practices.

### Study population

We adopted a previously described algorithm to identify MS cases.^1,15^ Briefly, we identified possible MS cases based on diagnostic and management primary care codes (Read codes), the International Classification of Diseases (ICD)-X codes and on prescription of disease-modifying therapies used exclusively to treat MS. Consistent with Culpepper et al.,^16^ to reduce the risk of misclassification, we defined MS cases as those with ⩾3 MS events recorded in their available clinical history. Date of the first MS diagnosis was considered the index date.^1^

As described elsewhere, additional inclusion criteria for MS cases were as follows: (1) diagnosis after 1 January 1987, when magnetic resonance imaging (MRI) was available to support the diagnosis; (2) continuous registration with the CPRD practice for ⩾1 year before the first MS event to ensure that information regarding key covariates was available at onset; (3) defined sex (male or female); (4) valid date of birth; (5) age ⩾18 years at cohort entry; (6) MS events recorded before the date of death; and (7) validity of patients’ clinical records in terms of continuous follow-up and data recording defined by the CPRD definition of up-to-standard (UTS).^1^ The UTS is deemed as the date at which the practice is considered to have high-quality data, based on continuity in data and death recording. Individuals were considered eligible if the clinical information recorded in the year before the index date and the follow-up were considered UTS.

PwMS were randomly matched to up to six people without MS by age, sex and general practice. Controls had UTS clinical data recorded during the study period and did not have MS or any other demyelinating disease event recorded (e.g. optic neuritis, transverse myelitis, acute disseminated encephalomyelitis and central nervous system demyelination not elsewhere classifiable); this minimized the possibility of including controls who might develop MS in the future. PwMS were matched to multiple controls to reduce the variance.^17^ We assigned the controls the index date of their matched MS case.

### Study variables

We extracted information on study variables at index year. Consistent with previous research using CPRD data,^18^ we defined study variables using comprehensive primary care code lists (which included both diagnostic and management codes) and ICD-X codes. This broader approach has been recommended in a previous validation study using CPRD data.^19^ Prescribing data were extracted using the British National Formulary (BNF) codes. Study outcomes included diagnosis of diabetes and hypertension, body mass index (BMI), systolic and diastolic blood pressure, treatment with lipid-lowering, oral antidiabetic and antihypertensive treatments (Supplemental Appendix Tables 1 and 2). Use of lipid-lowering medication was considered as proxy of dyslipidemia because the proportion of cholesterol levels recorded in this study population was too low.

Study covariates included the following socio-demographic characteristics: age (continuous), sex, ethnicity (white, non-white) and index of multiple deprivation (quintiles);^20^ vascular risk factors including smoking status (current smoker, former smoker, non-smoker) and antiplatelet treatment in the index year and year of MS diagnosis. Consistently with previously adopted methodology,^18^ study covariates were determined considering information available in primary care and hospital data (age, sex, ethnicity and smoking status), as well as linkage data (index of multiple deprivation).^13^ Information on antiplatelet treatment was extracted using BNF codes. We also included the number of primary care visits preceding the index year, to account for differences in health care utilization between the MS and matched cohorts (surveillance bias), and the eFI, a score which identifies people with frailty by including 36 equally weighted deficit variables using routinely collected primary care data (Supplemental Appendix Table 3).^21,22^ The eFI score was calculated considering the number of deficits identified divided by the total. Individuals were classified as fit (a score below 0.12), mildly frail (0.12 to 0.24), moderately frail (0.24 to 0.36) or severely frail (0.36 and above).^22^

### Statistical analysis

To reduce missing data at index year, we used the latest clinical data for each individual within the 5 years before the start of the study period.^1,15^ After checking missing data assumptions, we used multiple imputation by chained equations (10 copies) to estimate missing data for blood pressure and BMI (49.9% for blood pressure and 50% for BMI). Variables entered in the regression models included MS status (yes/no), sex, ethnicity, region, deprivation index, number of primary care visits in the previous year, smoking status, number of comorbidities (defined by a previously published list)^23^, treatment with lipid-lowering, oral antidiabetic, antiplatelet, anticoagulant and antihypertensive therapies in the index year.

Differences in study variables between PwMS and controls at the index year were assessed using the Chi-square, Student’s *t*-tests and the Kruskal–Wallis tests, as appropriate. To compare prevalence in PwMS and matched controls, we estimated the prevalence ratios (PRs) at baseline employing multivariable logistic regression models. Similarly, we employed linear regression models to estimate differences in the means of continuous outcomes (blood pressure, BMI). Multivariable regression models were adjusted for the study covariates indicated above. We repeated these analyses after stratifying by sex to assess effect modification.

#### Sensitivity analysis

Considering the percentage of missing data for blood pressure and BMI, we repeated the analyses limited to complete cases to check consistency with main analyses.

Results are presented as regression coefficients (coeff.), PRs and 95% confidence intervals (95% CIs), as appropriate. A *p*-value < 0.05 was considered statistically significant. We used Stata 17 MP (StataCorp. 2017, College Station, TX, USA: StataCorp LLC) to conduct statistical analyses.

## Results

### Study population

We identified 12,251 PwMS diagnosed between January 1987 and December 2018 and 72,572 matched controls. On average, each MS subject was matched to 5.9 (±0.3) controls. The average age at index (diagnosis) year was 44.9 years (±13.3), 70% of the population were female, and 20% of the population lived in deprived areas. The proportion of smokers was greater in PwMS than matched controls (37.9% vs 29.4%). On average, 3.8% of PwMS were at least mid-frail, 1.2% more than matched controls. PwMS had 2.7-fold the number of primary care visits as controls in the year preceding the index year (Table 1).

**Table 1.** Characteristics of the study population.

	Male			Female			Overall		
	MS subjects	Control subjects	*p*-value	MS subjects	Control subjects	*p*-value	MS subjects	Control subjects	*p*-value
*N*	3685	21,931		8566	50,640		12,251	72,572	
Follow-up time (years)	9.9 (6.1)	11.4 (6.5)	<0.001	10.4 (6.3)	11.5 (6.5)	<0.001	10.3 (6.3)	11.5 (6.5)	<0.001
Female (%)							69.9	69.8	0.752
Age (years)	46.3 (13.3)	46.3 (13.3)	0.852	44.3 (13.3)	44.3 (13.3)	0.907	44.9 (13.3)	44.9 (13.3)	0.727
Ethnicity – white (%)	92.3	93.5	0.013	91.5	94.1	<0.001	93.9	91.2	<0.001
Smoking status (%)									
Non-smoker	41.5	53.8		49.5	60.0		47.1	58.1	
Ex-smoker	17.5	14.7	<0.001	13.9	11.6	<0.001	15	12.5	<0.001
Current smoker	41.1	31.6		36.6	28.5		37.9	29.4	
eFI ratio	0.02 (0.04)	0.01 (0.03)	<0.001	0.03 (0.04)	0.02 (0.04)	<0.001	0.03 (0.04)	0.02 (0.04)	<0.001
Fit	97.2	98.3	<0.001	95.8	97.1	<0.001	96.2	97.4	<0.001
Mid frailty	2.8	1.6		4.0	2.8		3.7	2.5	
Moderate frailty	0.0	0.0		0.2	0.1		0.1	0.1	
Severe frailty	0.0	0.0		0.0	0.0		0.0	0.0	
Number of primary care visits in previous year	6.9 (10.3)	2.2 (5.0)	<0.001	8.2 (11.5)	3.2 (6.1)	<0.001	7.8 (11.2)	2.9 (5.9)	<0.001
Index of multiple deprivation (IMD; %)									
1Q – least deprived	13.7	13.7		14.6	14.6		14.4	14.4	
2Q	18.5	18.4	1.000	18.5	18.5	1.000	18.5	18.5	1.000
3Q	17.6	17.6		17.9	17.9		17.8	17.8	
4Q	20.2	20.3		18.8	18.8		19.2	19.2	
5Q – most deprived	20.6	20.6		20.3	20.3		20.4	20.4	
Missing data	9.3	9.4		9.8	9.9		9.7	9.7	

Individuals were classified as fit, if the eFI score was below 0.12; mildly frail, if the score was between 0.12 and 0.24; moderately frail, if the score was between 0.24 and 0.36; severely frail, if the score was 0.36 and above. Differences between groups were assessed employing Chi-square test, Student’s *t*-test and the Kruskal–Wallis test, as appropriate.

### Differences in diagnoses and medication usage

#### Type 2 diabetes

**Figure 1.**
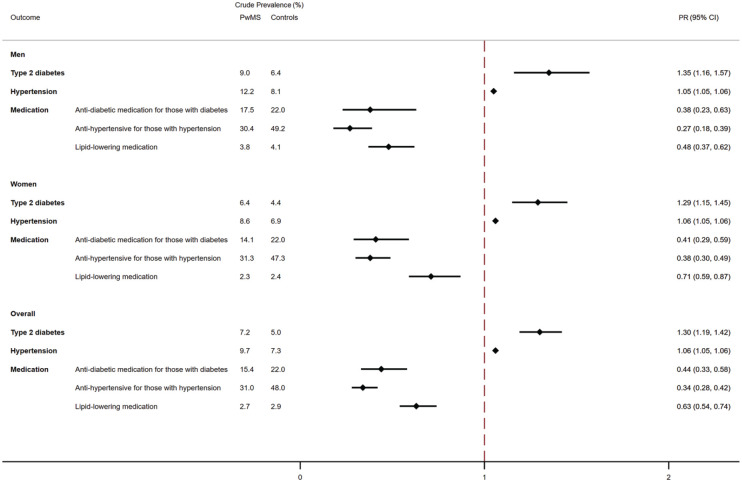
Adjusted proportions of diabetes and hypertension medication usage at index year. Crude prevalence for both People with Multiple Sclerosis (PwMS) and matched controls is reported in the columns on the left. For BMI categories, the value represents the proportion of those falling within the range. Adjusted prevalence ratios between PwMS and matched controls in the index year were estimated employing logistic regression models. Models were adjusted for gender, age, ethnicity (white/non-white), deprivation, smoking status, BMI, systolic and diastolic blood pressure, electronic frailty index (eFI) ratio, number of primary care visits in the year before and year. PwMS: People with Multiple Sclerosis; PR: prevalence ratio.

7.2% of PwMS had type 2 diabetes, compared to 5.0% of matched controls. After controlling for confounders, PwMS still had a 30% increased prevalence of type 2 diabetes at baseline, as compared with matched controls (PR = 1.30, 95% CI = 1.19, 1.42; Figure 1). The PR between PwMS and matched controls was greater in men (PR = 1.35; 95% CI = 1.16, 1.57) than in women (PR = 1.29, 95% CI = 1.15, 1.45), although the difference was not significant (*p* = 0.665). Among subjects with type 2 diabetes at index year (*n* = 4511), PwMS had a 56% lower prevalence of antidiabetic usage compared with controls (PR = 0.44, 95% CI = 0.33, 0.58). Stratifying by sex, the PR of antidiabetic usage was lower for men (PR = 0.38, 95% CI = 0.23, 0.63) than women (PR = 0.41, 95% CI = 0.29, 0.59), although the difference was not significant (*p* = 0.830).

#### Hypertension

Overall, 9.7% of PwMS had a diagnosis of hypertension as compared with 7.3% of matched controls. Although the difference between the cohorts was attenuated after controlling for confounders, the prevalence remained 6% higher (PR = 1.06, 95% CI = 1.05, 1.06). However, among those with a diagnosis of hypertension (*n* = 4817), PwMS had a 56% lower prevalence of antihypertensive usage at index year (PR = 0.34, 95% CI = 0.28, 0.42). The PR was even lower in men (PR = 0.27, 95% CI = 0.18, 0.39) than in women (PR = 0.38, 95% CI = 0.30, 0.49; Figure 1), but the difference was not statistically significant (*p* = 0.097).

#### Hyperlipidaemia

Treatment with lipid-lowering medications was lower in PwMS, as compared with matched controls (PR = 0.63, 95% CI = 0.54, 0.74). This was particularly pronounced for men (women: PR = 0.71, 95% CI = 0.59, 0.87; men: PR = 0.41, 95% CI = 0.37, 0.62).

### Differences in risk factor severity

As compared with matched controls, after adjustment, PwMS had a 0.4-mm Hg lower *systolic* blood pressure at the index year. The magnitude was greater for men than women, considering that men had almost a 3-mm Hg lower blood pressure than matched controls (overall: coeff. = −0.37, 95% CI = −0.60, −0.13; women: −0.54, 95% CI = −0.96, −0.12; men: −2.81, 95% CI = −3.84, −1.77). The differences were greater when restricting analyses to only those with a diagnosis of hypertension at baseline, as PwMS had a 3.3-mm Hg lower systolic blood pressure then matched controls (coeff. = −3.27, 95% CI = −5.04, −1.50); differences were confirmed in women but not in men (women: coeff. = −2.56, 95% CI = −3.84, −1.27; men: −0.27, 95% CI = 0.01, −2.56).

In contrast, PwMS had higher levels of *diastolic* blood pressure at baseline, as compared with matched controls (coeff. = 0.29, 95% CI = 0.14, 0.43). However, the differences were not confirmed when restricting analyses to only those with hypertension at baseline. Sex-stratified analyses for diastolic blood pressure showed opposing findings for men and women, with finding for the latter group being consistent with those of the general population. Men had a 0.7 mm Hg lower diastolic blood pressure (coeff. = −0.66, 95% CI = −1.28, −0.04), as compared with matched controls but men with a diagnosis of hypertension at baseline had 0.3 mm Hg higher diastolic blood pressure than controls (coeff. = 0.30, 95% CI = 0.13, 0.48).

**Figure 2.**
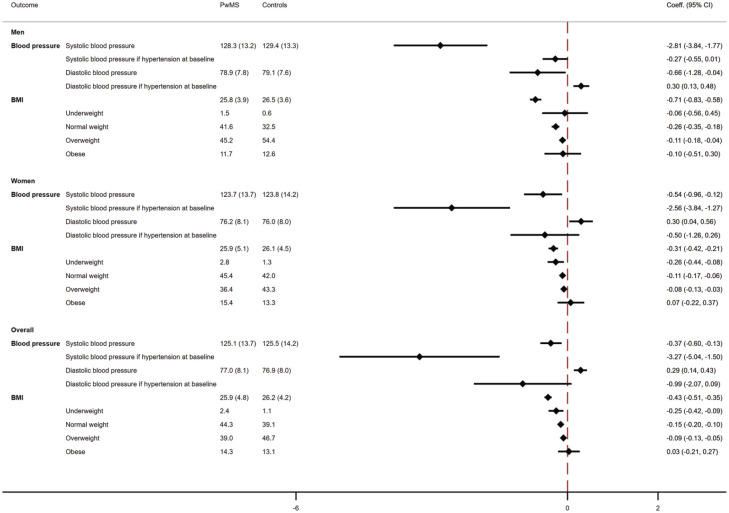
Adjusted differences in risk factors between MS and control subjects at index year. Adjusted differences in risk factors mean values in the index years were estimated employing linear regression models. Models were adjusted for gender, age, ethnicity (white/non-white), deprivation, smoking status, BMI, systolic and diastolic blood pressure, electronic frailty index (eFI) ratio, number of primary care visits in the year before and year. Columns on the left report unadjusted mean values at index year. PwMS: People with Multiple Sclerosis; coeff.: coefficient.

PwMS had lower levels of BMI at index year, as compared with matched controls (coeff. = −0.43, 95% CI = −0.51, −0.35). The differences were attenuated when progressing towards higher BMI ranges (underweight: coeff. = −0.25, 95% CI = −0.42, −0.09; normal weight: coeff. = −0.25, 95% CI = −0.42, −0.09; overweight: coeff. = −0.09, 95% CI = −0.13, −0.05; obese: coeff. = 0.03, 95% CI = −0.21, 0.27). Differences were confirmed when stratifying analyses by sex (Figure 2).

### Sensitivity analysis

Complete case analysis for differences in study outcomes at index year between PwMS and matched controls confirmed our main findings (Supplemental Appendix Table 4).

## Discussion

We conducted a large population-based study on 12,251 PwMS and 72,572 controls matched by age, sex and general practice between January 1987 and December 2018 in England to assess differences in the prevalence and management of vascular risk at the time of the diagnosis of MS as compared with the general population. We have found a 30% increased prevalence of diabetes in PwMS, but paradoxically, a 56% reduced likelihood of being treated with antidiabetic medication. Similarly, the prevalence of hypertension was 6% greater in PwMS, but the probability of being treated with antihypertensive medication was 56% lower. Importantly, for PwMS who had hypertension at time of diagnosis, even if the proportion of antihypertensive medication usage was lower than in matched controls, the actual systolic blood pressure values were, on average, lower in PwMS. This result was only partially confirmed in sex-stratified analyses, as findings had opposite directions for women and men, with analyses for men with MS showing higher prevalence of hypertension at diagnosis, lower proportion of antihypertensive medication usage among those with hypertension and higher diastolic blood pressure values than matched controls. PwMS also had a 37% lower probability of using lipid-lowering medication at the time of diagnosis. At time of diagnosis, BMI was 0.4 lower in PwMS as compared with controls. We found little or no difference between PwMS and matched controls when restricting analyses to those who were overweight or obese. Overall, results were confirmed when stratifying by sex.

The 7.2% prevalence of type 2 diabetes at diagnosis was consistent with a prior Canadian study which examined comorbidity prevalence at diagnosis (5.7%).^6^ The prevalence was lower than estimated in a prior meta-analysis (8.6%),^7^ which did not focus on prevalence at MS diagnosis specifically. Interestingly, the prevalence of hypertension at diagnosis (9.7%) was lower than that reported in the prior Canadian study (15.2%).^24^ This finding was also supported by the lower absolute systolic blood pressure values in PwMS than matched controls in this study. In contrast, no clinically meaningful differences in diastolic blood pressure values were found, consistent with recent findings which found no differences in temporal trends in the incidence of hypertension between PwMS and matched controls.^2^ Overall, BMI was lower in PwMS than matched controls and lower than estimates reported in previous study.^4,7,24^ However, we observed a significant proportion of PwMS to be underweight (2.4%) which would have reduced the average BMI.

Generally, the association between comorbid disease and intensity of management of vascular risk factors varies in magnitude and direction.^25,26^ We found that PwMS were less likely than matched controls to be treated if they had type 2 diabetes and hypertension. While the findings regarding type 2 diabetes are consistent with recent evidence,^4,27^ those regarding likelihood of being treated with antihypertensive medications, contradict recent evidence that found no difference.^4^ That study, however, did not focus on differences at diagnosis, and our findings might have differed if we had focused on prevalent cohorts *post-MS diagnosis* since PwMS have higher healthcare resource utilization following the diagnosis,^9^ which could lead to tighter clinical management following diagnosis. A growing body of evidence shows the benefits of this medication on disease progression in PwMS.^28,29^ Nonetheless, we found that PwMS were also less likely to be treated with statins, consistent with a Canadian study showing that PwMS were less likely to receive statins following admission for acute myocardial infarction.^30^

PwMS have an increased prevalence of comorbidities.^2^ Some studies suggest that in primary care, individuals with more unrelated comorbidities, such as arthritis, are less likely to have their uncontrolled hypertension addressed.^26,31^ Some, but not all, studies suggest that comorbidities that are unrelated or discordant with diabetes are associated with worse diabetes control.^32^ This may reflect clinician or patient priorities, competing demands and challenges with treatment adherence.

Sex differences in vascular risk and risk management are complex. At diagnosis, men had a higher prevalence of hypertension and diabetes than women in both populations, consistent with a prior Canadian study.^6^ Before the menopause, women in the general population have a lower prevalence of hypertension than men, and this association reverses post-menopause.^33^ Sex-specific differences in vascular risk management have been reported in some populations. Among individuals with coronary heart disease from Europe, Asian and the Middle East, women were less likely to reach targets for cholesterol and glucose than men, but were more likely to reach targets for blood pressure.^34^ In a Canadian MS cohort, women were less likely to exhibit good adherence to statins, angiotensin-converting enzyme (ACE) inhibitors and angiotensin receptor blockers than men.^35^

To our knowledge, this was the first study that controlled for important clinical variables, including blood pressure, BMI and frailty index when assessing vascular risk management at the time of the diagnosis of MS. We note the frailty index is strongly associated with MS disease duration, disability and fatigue.^12^ Moreover, frailty is associated with higher prevalence of hypertension and worse hypertension control,^36,37^ as well as worse cardiovascular outcomes.^38^

Several caveats merit discussion. First, when using routinely collected data, miscoding, misclassification and misdiagnosis may occur. However, the CPRD is a reliable, widely used data source and is subject to regular quality checks.^13^ Second, PwMS diagnosed before availability of Disease Modifying Therapies in the United Kingdom (1995) may have been more likely to be exposed to corticosteroid treatment, with subsequent negative impact on their vascular risk profile. However, only 4.6% of the PwMS in this study population had a diagnosis of MS before 1995; therefore, the impact on our findings might be limited. Third, although CPRD is a database representative of the UK population and although we adopted a validated algorithm to identify PwMS, we cannot be completely certain if the MS population identified using our case-finding algorithm is fully representative of all people with MS in England. Fourth, we used lipid-lowering medication as proxy of dyslipidemia because we lacked sufficient data regarding cholesterol levels which may have reduced ascertainment of dyslipidemia. Fifth, we could not assess any non-pharmacologic recommendations, such as changes in diet or physical activity, that might have been made to manage vascular risk. Finally, this is a cross-sectional study which limits causal inference regarding our findings.

In summary, at the time of diagnosis, PwMS have an increased prevalence of vascular risk factors, including hypertension and diabetes, although paradoxically poorer treatment, with probabilities of initiating treatment being around 40%–60% less than matched controls. This is concerning, because we know that in PwMS the vascular burden increases over time^2^ and is associated with accelerated MS-related disability,^8^ increased healthcare utilization^9^ and greater all-cause and vascular mortality.^1,3^ Further research is needed to determine the optimal approach to vascular risk management in this population and to develop appropriate guidelines to guide clinical practice.

## Supplemental Material

sj-docx-1-msj-10.1177_13524585231164296 – Supplemental material for Management of vascular risk in people with multiple sclerosis at the time of diagnosis in England: A population-based study Click here for additional data file. Supplemental material, sj-docx-1-msj-10.1177_13524585231164296 for Management of vascular risk in people with multiple sclerosis at the time of diagnosis in England: A population-based study by Raffaele Palladino, Ruth Ann Marrie, Azeem Majeed and Jeremy Chataway in Multiple Sclerosis Journal
