# Supplementary material for: Management of vascular risk in people with multiple sclerosis at the time of diagnosis in England: A population-based study
Source: Mult Scler. 2023 Apr 7;29(6):671–9. doi: 10.1177/13524585231164296 (PMC10176618; doi:10.1177/13524585231164296)
Supplement: sj-docx-1-msj-10.1177_13524585231164296 – Supplemental material for Management of vascular risk in people with multiple sclerosis at the time of diagnosis in England: A population-based study [file sj-docx-1-msj-10.1177_13524585231164296.docx]

**Management of vascular risk in people with multiple sclerosis at the time of diagnosis in England: a population-based study**

Raffaele Palladino MD^1,2^, Ruth Ann Marrie MD, PhD^3^, Azeem Majeed MD^1^, Jeremy Chataway PhD, FRCP^4,5^

1. Department of Primary Care and Public Health, School of Public Health, Imperial College of London, London, United Kingdom.

2. Department of Public Health, Federico II University, Naples, Italy

3. Departments of Medicine and Community Health Sciences, Max Rady College of Medicine, Rady Faculty of Health Sciences, University of Manitoba, Winnipeg, MB, Canada,

4. Queen Square Multiple Sclerosis Centre, Department of Neuroinflammation, UCL Queen Square Institute of Neurology, Faculty of Brain Sciences, University College London, London, United Kingdom

5.National Institute for Health Research, University College London Hospitals, Biomedical Research Centre, London, United Kingdom

**Appendix Table 1:** Code list for study outcomes

| **Type 2 Diabetes** | | | |
| --- | --- | --- | --- |
| **Read code list** | | | |
| **Medcode** | **Readcode** | **Readterm** | |
| 7563 | 66A3.00 | Diabetic on diet only | |
| 1684 | 66A4.00 | Diabetic on oral treatment | |
| 1684 | 66A4.00 | Diabetic on oral treatment | |
| 107508 | 66AH200 | Conversion to insulin by diabetes specialist nurse | |
| 83532 | 66Ao.00 | Diabetes type 2 review | |
| 101801 | 66At100 | Type II diabetic dietary review | |
| 102611 | 66At111 | Type 2 diabetic dietary review | |
| 93657 | 8Hj4.00 | Referral to DESMOND diabetes structured education programme | |
| 95159 | 9NiD.00 | Did not attend DESMOND diabetes structured education program | |
| 93529 | 9OLK.00 | DESMOND diabetes structured education programme completed | |
| 14803 | C100100 | Diabetes mellitus, adult onset, no mention of complication | |
| 506 | C100112 | Non-insulin dependent diabetes mellitus | |
| 43139 | C102100 | Diabetes mellitus, adult onset, with hyperosmolar coma | |
| 35105 | C104100 | Diabetes mellitus, adult onset, with renal manifestation | |
| 41389 | C105100 | Diabetes mellitus, adult onset, + ophthalmic manifestation | |
| 39317 | C106100 | Diabetes mellitus, adult onset, + neurological manifestation | |
| 63357 | C107100 | Diabetes mellitus, adult, + peripheral circulatory disorder | |
| 33807 | C107200 | Diabetes mellitus, adult with gangrene | |
| 56803 | C107400 | NIDDM with peripheral circulatory disorder | |
| 4513 | C109.00 | Non-insulin dependent diabetes mellitus | |
| 5884 | C109.11 | NIDDM - Non-insulin dependent diabetes mellitus | |
| 17859 | C109.12 | Type 2 diabetes mellitus | |
| 18219 | C109.13 | Type II diabetes mellitus | |
| 52303 | C109000 | Non-insulin-dependent diabetes mellitus with renal comps | |
| 50225 | C109011 | Type II diabetes mellitus with renal complications | |
| 18209 | C109012 | Type 2 diabetes mellitus with renal complications | |
| 50429 | C109100 | Non-insulin-dependent diabetes mellitus with ophthalm comps | |
| 59725 | C109111 | Type II diabetes mellitus with ophthalmic complications | |
| 70316 | C109112 | Type 2 diabetes mellitus with ophthalmic complications | |
| 55842 | C109200 | Non-insulin-dependent diabetes mellitus with neuro comps | |
| 67905 | C109211 | Type II diabetes mellitus with neurological complications | |
| 45919 | C109212 | Type 2 diabetes mellitus with neurological complications | |
| 62146 | C109300 | Non-insulin-dependent diabetes mellitus with multiple comps | |
| 108005 | C109312 | Type 2 diabetes mellitus with multiple complications | |
| 34912 | C109400 | Non-insulin dependent diabetes mellitus with ulcer | |
| 55075 | C109411 | Type II diabetes mellitus with ulcer | |
| 65704 | C109412 | Type 2 diabetes mellitus with ulcer | |
| 40401 | C109500 | Non-insulin dependent diabetes mellitus with gangrene | |
| 62107 | C109511 | Type II diabetes mellitus with gangrene | |
| 46150 | C109512 | Type 2 diabetes mellitus with gangrene | |
| 17262 | C109600 | Non-insulin-dependent diabetes mellitus with retinopathy | |
| 58604 | C109611 | Type II diabetes mellitus with retinopathy | |
| 42762 | C109612 | Type 2 diabetes mellitus with retinopathy | |
| 8403 | C109700 | Non-insulin dependent diabetes mellitus - poor control | |
| 24458 | C109711 | Type II diabetes mellitus - poor control | |
| 45913 | C109712 | Type 2 diabetes mellitus - poor control | |
| 29979 | C109900 | Non-insulin-dependent diabetes mellitus without complication | |
| 105784 | C109912 | Type 2 diabetes mellitus without complication | |
| 72320 | C109A00 | Non-insulin dependent diabetes mellitus with mononeuropathy | |
| 50813 | C109A11 | Type II diabetes mellitus with mononeuropathy | |
| 45467 | C109B00 | Non-insulin dependent diabetes mellitus with polyneuropathy | |
| 47409 | C109B11 | Type II diabetes mellitus with polyneuropathy | |
| 59365 | C109C00 | Non-insulin dependent diabetes mellitus with nephropathy | |
| 64571 | C109C11 | Type II diabetes mellitus with nephropathy | |
| 24836 | C109C12 | Type 2 diabetes mellitus with nephropathy | |
| 43785 | C109D00 | Non-insulin dependent diabetes mellitus with hypoglyca coma | |
| 56268 | C109D11 | Type II diabetes mellitus with hypoglycaemic coma | |
| 61071 | C109D12 | Type 2 diabetes mellitus with hypoglycaemic coma | |
| 69278 | C109E00 | Non-insulin depend diabetes mellitus with diabetic cataract | |
| 48192 | C109E11 | Type II diabetes mellitus with diabetic cataract | |
| 44779 | C109E12 | Type 2 diabetes mellitus with diabetic cataract | |
| 54212 | C109F00 | Non-insulin-dependent d m with peripheral angiopath | |
| 54899 | C109F11 | Type II diabetes mellitus with peripheral angiopathy | |
| 60699 | C109F12 | Type 2 diabetes mellitus with peripheral angiopathy | |
| 24693 | C109G00 | Non-insulin dependent diabetes mellitus with arthropathy | |
| 18143 | C109G11 | Type II diabetes mellitus with arthropathy | |
| 49869 | C109G12 | Type 2 diabetes mellitus with arthropathy | |
| 40962 | C109H00 | Non-insulin dependent d m with neuropathic arthropathy | |
| 47816 | C109H11 | Type II diabetes mellitus with neuropathic arthropathy | |
| 66965 | C109H12 | Type 2 diabetes mellitus with neuropathic arthropathy | |
| 18278 | C109J00 | Insulin treated Type 2 diabetes mellitus | |
| 37648 | C109J11 | Insulin treated non-insulin dependent diabetes mellitus | |
| 18264 | C109J12 | Insulin treated Type II diabetes mellitus | |
| 36633 | C109K00 | Hyperosmolar non-ketotic state in type 2 diabetes mellitus | |
| 758 | C10F.00 | Type 2 diabetes mellitus | |
| 22884 | C10F.11 | Type II diabetes mellitus | |
| 18777 | C10F000 | Type 2 diabetes mellitus with renal complications | |
| 57278 | C10F011 | Type II diabetes mellitus with renal complications | |
| 47321 | C10F100 | Type 2 diabetes mellitus with ophthalmic complications | |
| 100964 | C10F111 | Type II diabetes mellitus with ophthalmic complications | |
| 34268 | C10F200 | Type 2 diabetes mellitus with neurological complications | |
| 98616 | C10F211 | Type II diabetes mellitus with neurological complications | |
| 65267 | C10F300 | Type 2 diabetes mellitus with multiple complications | |
| 43227 | C10F311 | Type II diabetes mellitus with multiple complications | |
| 49074 | C10F400 | Type 2 diabetes mellitus with ulcer | |
| 91646 | C10F411 | Type II diabetes mellitus with ulcer | |
| 12736 | C10F500 | Type 2 diabetes mellitus with gangrene | |
| 104323 | C10F511 | Type II diabetes mellitus with gangrene | |
| 18496 | C10F600 | Type 2 diabetes mellitus with retinopathy | |
| 49655 | C10F611 | Type II diabetes mellitus with retinopathy | |
| 25627 | C10F700 | Type 2 diabetes mellitus - poor control | |
| 47315 | C10F711 | Type II diabetes mellitus - poor control | |
| 47954 | C10F900 | Type 2 diabetes mellitus without complication | |
| 53392 | C10F911 | Type II diabetes mellitus without complication | |
| 62674 | C10FA00 | Type 2 diabetes mellitus with mononeuropathy | |
| 95351 | C10FA11 | Type II diabetes mellitus with mononeuropathy | |
| 18425 | C10FB00 | Type 2 diabetes mellitus with polyneuropathy | |
| 50527 | C10FB11 | Type II diabetes mellitus with polyneuropathy | |
| 12640 | C10FC00 | Type 2 diabetes mellitus with nephropathy | |
| 102201 | C10FC11 | Type II diabetes mellitus with nephropathy | |
| 46917 | C10FD00 | Type 2 diabetes mellitus with hypoglycaemic coma | |
| 98723 | C10FD11 | Type II diabetes mellitus with hypoglycaemic coma | |
| 44982 | C10FE00 | Type 2 diabetes mellitus with diabetic cataract | |
| 44982 | C10FE00 | Type 2 diabetes mellitus with diabetic cataract | |
| 93727 | C10FE11 | Type II diabetes mellitus with diabetic cataract | |
| 37806 | C10FF00 | Type 2 diabetes mellitus with peripheral angiopathy | |
| 104639 | C10FF11 | Type II diabetes mellitus with peripheral angiopathy | |
| 59253 | C10FG00 | Type 2 diabetes mellitus with arthropathy | |
| 103902 | C10FG11 | Type II diabetes mellitus with arthropathy | |
| 35385 | C10FH00 | Type 2 diabetes mellitus with neuropathic arthropathy | |
| 1407 | C10FJ00 | Insulin treated Type 2 diabetes mellitus | |
| 64668 | C10FJ11 | Insulin treated Type II diabetes mellitus | |
| 34450 | C10FK00 | Hyperosmolar non-ketotic state in type 2 diabetes mellitus | |
| 107701 | C10FK11 | Hyperosmolar non-ketotic state in type II diabetes mellitus | |
| 107701 | C10FK11 | Hyperosmolar non-ketotic state in type II diabetes mellitus | |
| 26054 | C10FL00 | Type 2 diabetes mellitus with persistent proteinuria | |
| 60796 | C10FL11 | Type II diabetes mellitus with persistent proteinuria | |
| 18390 | C10FM00 | Type 2 diabetes mellitus with persistent microalbuminuria | |
| 18390 | C10FM00 | Type 2 diabetes mellitus with persistent microalbuminuria | |
| 85991 | C10FM11 | Type II diabetes mellitus with persistent microalbuminuria | |
| 32627 | C10FN00 | Type 2 diabetes mellitus with ketoacidosis | |
| 106528 | C10FN11 | Type II diabetes mellitus with ketoacidosis | |
| 51756 | C10FP00 | Type 2 diabetes mellitus with ketoacidotic coma | |
| 106061 | C10FP11 | Type II diabetes mellitus with ketoacidotic coma | |
| 25591 | C10FQ00 | Type 2 diabetes mellitus with exudative maculopathy | |
| 63690 | C10FR00 | Type 2 diabetes mellitus with gastroparesis | |
| 11047 | 66AH000 | Conversion to insulin | |
| 107331 | 66AH100 | Conversion to insulin in secondary care | |
| 108018 | 66o0.00 | Incretin mimetic treatment started | |
| 95093 | 8I83.00 | Did not complete DESMOND diabetes structured educat program | |
| 103543 | 8IE9.00 | Referral to DESMOND structured programme declined | |
| 93530 | 9OLE.00 | Attended DESMOND structured programme | |
| 21689 | 13AB.00 | Diabetic lipid lowering diet | |
| 13078 | 13AC.00 | Diabetic weight reducing diet | |
| 13074 | 13B1.00 | Diabetic diet | |
| 6813 | 1434 | H/O: diabetes mellitus | |
| 22967 | 2BBF.00 | Retinal abnormality - diabetes related | |
| 13100 | 2BBJ.00 | O/E - no right diabetic retinopathy | |
| 13104 | 2BBK.00 | O/E - no left diabetic retinopathy | |
| 47328 | 2BBk.00 | O/E - right eye stable treated prolif diabetic retinopathy | |
| 9835 | 2BBL.00 | O/E - diabetic maculopathy present both eyes | |
| 52041 | 2BBl.00 | O/E - left eye stable treated prolif diabetic retinopathy | |
| 47144 | 2BBM.00 | O/E - diabetic maculopathy absent both eyes | |
| 52630 | 2BBo.00 | O/E - sight threatening diabetic retinopathy | |
| 13099 | 2BBR.00 | O/E - right eye preproliferative diabetic retinopathy | |
| 101881 | 2BBr.00 | Impaired vision due to diabetic retinopathy | |
| 13103 | 2BBS.00 | O/E - left eye preproliferative diabetic retinopathy | |
| 13097 | 2BBT.00 | O/E - right eye proliferative diabetic retinopathy | |
| 13101 | 2BBV.00 | O/E - left eye proliferative diabetic retinopathy | |
| 13102 | 2BBW.00 | O/E - right eye diabetic maculopathy | |
| 13108 | 2BBX.00 | O/E - left eye diabetic maculopathy | |
| 17095 | 2G5A.00 | O/E - Right diabetic foot at risk | |
| 26664 | 2G5B.00 | O/E - Left diabetic foot at risk | |
| 18056 | 2G5C.00 | Foot abnormality - diabetes related | |
| 105740 | 2G5d.00 | O/E - Left diabetic foot at increased risk | |
| 26666 | 2G5E.00 | O/E - Right diabetic foot at low risk | |
| 105741 | 2G5e.00 | O/E - Right diabetic foot at increased risk | |
| 31157 | 2G5F.00 | O/E - Right diabetic foot at moderate risk | |
| 31171 | 2G5G.00 | O/E - Right diabetic foot at high risk | |
| 35316 | 2G5H.00 | O/E - Right diabetic foot - ulcerated | |
| 26667 | 2G5I.00 | O/E - Left diabetic foot at low risk | |
| 31156 | 2G5J.00 | O/E - Left diabetic foot at moderate risk | |
| 31172 | 2G5K.00 | O/E - Left diabetic foot at high risk | |
| 35116 | 2G5L.00 | O/E - Left diabetic foot - ulcerated | |
| 62384 | 2G5V.00 | O/E - right chronic diabetic foot ulcer | |
| 49640 | 2G5W.00 | O/E - left chronic diabetic foot ulcer | |
| 34528 | 3882 | Diabetes well being questionnaire | |
| 98954 | 3883 | Diabetes treatment satisfaction questionnaire | |
| 14050 | 42c..00 | HbA1 - diabetic control | |
| 9958 | 42W..00 | Hb. A1C - diabetic control | |
| 14049 | 42WZ.00 | Hb. A1C - diabetic control NOS | |
| 108993 | 661M400 | Diabetes self-management plan agreed | |
| 107423 | 661N400 | Diabetes self-management plan review | |
| 3550 | 66A..00 | Diabetic monitoring | |
| 13070 | 66A1.00 | Initial diabetic assessment | |
| 608 | 66A2.00 | Follow-up diabetic assessment | |
| 8842 | 66A5.00 | Diabetic on insulin | |
| 13069 | 66A8.00 | Has seen dietician - diabetes | |
| 38078 | 66A9.00 | Understands diet - diabetes | |
| 25636 | 66Aa.00 | Diabetic diet - poor compliance | |
| 22823 | 66Ab.00 | Diabetic foot examination | |
| 10977 | 66Ac.00 | Diabetic peripheral neuropathy screening | |
| 13196 | 66AD.00 | Fundoscopy - diabetic check | |
| 32619 | 66Af.00 | Patient diabetes education review | |
| 53238 | 66AG.00 | Diabetic drug side effects | |
| 16490 | 66AH.00 | Diabetic treatment changed | |
| 13071 | 66AI.00 | Diabetic - good control | |
| 28873 | 66Ai.00 | Diabetic 6 month review | |
| 2378 | 66AJ.00 | Diabetic - poor control | |
| 9013 | 66AJ.11 | Unstable diabetes | |
| 22023 | 66AJz00 | Diabetic - poor control NOS | |
| 43951 | 66AK.00 | Diabetic - cooperative patient | |
| 66475 | 66Ak.00 | Diabetic monitoring - lower risk albumin excretion | |
| 17869 | 66AL.00 | Diabetic-uncooperative patient | |
| 61470 | 66Al.00 | Diabetic monitoring - higher risk albumin excretion | |
| 17886 | 66AM.00 | Diabetic - follow-up default | |
| 29041 | 66AN.00 | Date diabetic treatment start | |
| 12506 | 66AP.00 | Diabetes: practice programme | |
| 12675 | 66AQ.00 | Diabetes: shared care programme | |
| 95994 | 66Aq.00 | Diabetic foot screen | |
| 8836 | 66AR.00 | Diabetes management plan given | |
| 6125 | 66AS.00 | Diabetic annual review | |
| 101728 | 66As.00 | Diabetic on subcutaneous treatment | |
| 107464 | 66AS000 | Diabetes Year of Care annual review | |
| 18167 | 66AT.00 | Annual diabetic blood test | |
| 101177 | 66At.00 | Diabetic dietary review | |
| 12307 | 66AU.00 | Diabetes care by hospital only | |
| 102434 | 66Au.00 | Diabetic erectile dysfunction review | |
| 28769 | 66AV.00 | Diabetic on insulin and oral treatment | |
| 102490 | 66Av.00 | Diabetic assessment of erectile dysfunction | |
| 50175 | 66AW.00 | Diabetic foot risk assessment | |
| 26604 | 66AY.00 | Diabetic diet - good compliance | |
| 13067 | 66AZ.00 | Diabetic monitoring NOS | |
| 107452 | 66o..00 | Further diabetic monitoring | |
| 12682 | 679R.00 | Patient offered diabetes structured education programme | |
| 104374 | 67D8.00 | Provision of diabetes clinical summary | |
| 18311 | 68A7.00 | Diabetic retinopathy screening | |
| 19739 | 68A9.00 | Diabetic retinopathy screening offered | |
| 61021 | 68AB.00 | Diabetic digital retinopathy screening offered | |
| 11599 | 7276 | Pan retinal photocoagulation for diabetes | |
| 47341 | 8A12.00 | Diabetic crisis monitoring | |
| 24363 | 8A13.00 | Diabetic stabilisation | |
| 11471 | 8B3l.00 | Diabetes medication review | |
| 12213 | 8BL2.00 | Patient on maximal tolerated therapy for diabetes | |
| 8414 | 8CA4100 | Pt advised re diabetic diet | |
| 105585 | 8CMW700 | Diabetes clinical pathway | |
| 63412 | 8CR2.00 | Diabetes clinical management plan | |
| 47032 | 8CS0.00 | Diabetes care plan agreed | |
| 7059 | 8H2J.00 | Admit diabetic emergency | |
| 35321 | 8H3O.00 | Non-urgent diabetic admission | |
| 94330 | 8H4e.00 | Referral to diabetes special interest general practitioner | |
| 12225 | 8H7C.00 | Refer, diabetic liaison nurse | |
| 8306 | 8H7f.00 | Referral to diabetes nurse | |
| 11677 | 8H7r.00 | Refer to diabetic foot screener | |
| 11018 | 8HBG.00 | Diabetic retinopathy 12 month review | |
| 18662 | 8HBH.00 | Diabetic retinopathy 6 month review | |
| 57723 | 8HHy.00 | Referral to diabetic register | |
| 47011 | 8Hj0.00 | Referral to diabetes structured education programme | |
| 64142 | 8Hl1.00 | Referral for diabetic retinopathy screening | |
| 82474 | 8Hl4.00 | Referral to community diabetes specialist nurse | |
| 104287 | 8Hlc.00 | Referral to community diabetes service | |
| 105207 | 8HTE100 | Referral to community diabetes clinic | |
| 69163 | 8HTi.00 | Referral to multidisciplinary diabetic clinic | |
| 19381 | 8HTk.00 | Referral to diabetic eye clinic | |
| 18824 | 8I3W.00 | Diabetic foot examination declined | |
| 12262 | 8I3X.00 | Diabetic retinopathy screening refused | |
| 12247 | 8I6G.00 | Diabetic foot examination not indicated | |
| 107414 | 8I94.00 | Diabetes structured education programme not available | |
| 101456 | 8IAs.00 | Diabetic dietary review declined | |
| 106679 | 8OA3.00 | Provision of written information about diabetes and driving | |
| 106269 | 9m0..00 | Diabetic retinopathy screening administrative status | |
| 106332 | 9m00.00 | Eligible for diabetic retinopathy screening | |
| 106218 | 9m0A.00 | Declined diabetic retinopathy screening | |
| 38103 | 9N0m.00 | Seen in diabetic nurse consultant clinic | |
| 32739 | 9N0n.00 | Seen in community diabetes specialist clinic | |
| 38129 | 9N0o.00 | Seen in community diabetic specialist nurse clinic | |
| 10824 | 9N1i.00 | Seen in diabetic foot clinic | |
| 95813 | 9N1o.00 | Seen in multidisciplinary diabetic clinic | |
| 2379 | 9N1Q.00 | Seen in diabetic clinic | |
| 12507 | 9N2i.00 | Seen by diabetic liaison nurse | |
| 9145 | 9N4I.00 | DNA - Did not attend diabetic clinic | |
| 30648 | 9N4p.00 | Did not attend diabetic retinopathy clinic | |
| 102768 | 9NiZ.00 | Did not attend diabetes foot screening | |
| 11930 | 9NN9.00 | Under care of diabetes specialist nurse | |
| 11094 | 9NND.00 | Under care of diabetic foot screener | |
| 9897 | 9OL..00 | Diabetes monitoring admin. | |
| 13191 | 9OL..11 | Diabetes clinic administration | |
| 13197 | 9OL1.00 | Attends diabetes monitoring | |
| 26603 | 9OL2.00 | Refuses diabetes monitoring | |
| 22130 | 9OL3.00 | Diabetes monitoring default | |
| 13194 | 9OL4.00 | Diabetes monitoring 1st letter | |
| 13195 | 9OL5.00 | Diabetes monitoring 2nd letter | |
| 12030 | 9OL6.00 | Diabetes monitoring 3rd letter | |
| 31240 | 9OL7.00 | Diabetes monitor.verbal invite | |
| 31141 | 9OL8.00 | Diabetes monitor.phone invite | |
| 13192 | 9OLA.00 | Diabetes monitor. check done | |
| 20900 | 9OLA.11 | Diabetes monitored | |
| 26605 | 9OLB.00 | Attended diabetes structured education programme | |
| 35383 | 9OLD.00 | Diabetic patient unsuitable for digital retinal photography | |
| 94186 | 9OLF.00 | Diabetes structured education programme completed | |
| 93854 | 9OLM.00 | Diabetes structured education programme declined | |
| 101455 | 9OLN.00 | Diabetes monitor invitation by SMS (short message service) | |
| 31241 | 9OLZ.00 | Diabetes monitoring admin.NOS | |
| 94647 | 9Oy..00 | Diabetes screening administration | |
| 106738 | 9Oy0000 | Diabetic foot screening invitation | |
| 106723 | 9Oy0200 | Diabetic foot screening invitation first letter | |
| 106722 | 9Oy0300 | Diabetic foot screening invitation second letter | |
| 107793 | 9Oy0400 | Diabetic foot screening invitation third letter | |
| 711 | C10..00 | Diabetes mellitus | |
| 38986 | C100.00 | Diabetes mellitus with no mention of complication | |
| 54856 | C101100 | Diabetes mellitus, adult onset, with ketoacidosis | |
| 21482 | C102.00 | Diabetes mellitus with hyperosmolar coma | |
| 16502 | C104.00 | Diabetes mellitus with renal manifestation | |
| 2475 | C104.11 | Diabetic nephropathy | |
| 35107 | C104z00 | Diabetes mellitus with nephropathy NOS | |
| 16230 | C106.00 | Diabetes mellitus with neurological manifestation | |
| 7795 | C106.12 | Diabetes mellitus with neuropathy | |
| 16491 | C106.13 | Diabetes mellitus with polyneuropathy | |
| 22573 | C106z00 | Diabetes mellitus NOS with neurological manifestation | |
| 35399 | C107.00 | Diabetes mellitus with peripheral circulatory disorder | |
| 35399 | C107.00 | Diabetes mellitus with peripheral circulatory disorder | |
| 32403 | C107.11 | Diabetes mellitus with gangrene | |
| 32556 | C107.12 | Diabetes with gangrene | |
| 65025 | C107z00 | Diabetes mellitus NOS with peripheral circulatory disorder | |
| 109103 | C109911 | Type II diabetes mellitus without complication | |
| 109865 | C109B12 | Type 2 diabetes mellitus with polyneuropathy | |
| 46624 | C10C.11 | Maturity onset diabetes in youth | |
| 36695 | C10D.00 | Diabetes mellitus autosomal dominant type 2 | |
| 59991 | C10D.11 | Maturity onset diabetes in youth type 2 | |
| 109197 | C10FH11 | Type II diabetes mellitus with neuropathic arthropathy | |
| 56885 | C10K000 | Type A insulin resistance without complication | |
| 94383 | C10N000 | Secondary diabetes mellitus without complication | |
| 107603 | C10P.00 | Diabetes mellitus in remission | |
| 107824 | C10P100 | Type II diabetes mellitus in remission | |
| 63371 | C10y100 | Diabetes mellitus, adult, + other specified manifestation | |
| 45491 | C10z.00 | Diabetes mellitus with unspecified complication | |
| 45491 | C10z.00 | Diabetes mellitus with unspecified complication | |
| 63762 | C10z100 | Diabetes mellitus, adult onset, + unspecified complication | |
| 52212 | Cyu2.00 | [X]Diabetes mellitus | |
| 17067 | F171100 | Autonomic neuropathy due to diabetes | |
| 44033 | F345000 | Diabetic mononeuritis multiplex | |
| 17247 | F35z000 | Diabetic mononeuritis NOS | |
| 31790 | F372.00 | Polyneuropathy in diabetes | |
| 5002 | F372.11 | Diabetic polyneuropathy | |
| 2342 | F372.12 | Diabetic neuropathy | |
| 48078 | F372000 | Acute painful diabetic neuropathy | |
| 35785 | F372100 | Chronic painful diabetic neuropathy | |
| 24571 | F372200 | Asymptomatic diabetic neuropathy | |
| 2340 | F381311 | Diabetic amyotrophy | |
| 37315 | F3y0.00 | Diabetic mononeuropathy | |
| 3286 | F420100 | Proliferative diabetic retinopathy | |
| 2986 | F420200 | Preproliferative diabetic retinopathy | |
| 10099 | F420300 | Advanced diabetic maculopathy | |
| 3837 | F420400 | Diabetic maculopathy | |
| 10755 | F420600 | Non proliferative diabetic retinopathy | |
| 30477 | F420700 | High risk proliferative diabetic retinopathy | |
| 65463 | F420800 | High risk non proliferative diabetic retinopathy | |
| 11626 | F420z00 | Diabetic retinopathy NOS | |
| 17313 | F440700 | Diabetic iritis | |
| 10659 | F464000 | Diabetic cataract | |
| 34152 | G73y000 | Diabetic peripheral angiopathy | |
| 2471 | K01x100 | Nephrotic syndrome in diabetes mellitus | |
| 105302 | K08yA00 | Proteinuric diabetic nephropathy | |
| 107881 | K08yA11 | Clinical diabetic nephropathy | |
| 106360 | K27y700 | Erectile dysfunction due to diabetes mellitus | |
| 7328 | M037200 | Cellulitis in diabetic foot | |
| 24327 | M271000 | Ischaemic ulcer diabetic foot | |
| 11663 | M271100 | Neuropathic diabetic ulcer - foot | |
| 9881 | M271200 | Mixed diabetic ulcer - foot | |
| 18142 | N030000 | Diabetic cheiroarthropathy | |
| 27891 | N030100 | Diabetic Charcot arthropathy | |
| 53634 | R054200 | [D]Gangrene of toe in diabetic | |
| 31053 | R054300 | [D]Widespread diabetic foot gangrene | |
| 10642 | ZC2C800 | Dietary advice for diabetes mellitus | |
| 45250 | ZL22500 | Under care of diabetic liaison nurse | |
| 11977 | ZL62500 | Referral to diabetes nurse | |
| 13678 | ZL62600 | Referral to diabetic liaison nurse | |
| 8618 | ZLA2500 | Seen by diabetic liaison nurse | |
| 68546 | ZRB4.00 | Diabetes clinic satisfaction questionnaire | |
| 38130 | ZRB6.00 | Diabetes wellbeing questionnaire |  |
| 16881 | ZV65312 | [V]Dietary counselling in diabetes mellitus |  |
|  |  | |  |
| **Entity codes** | | |  |
| 18 | Diabetic register | |  |
| 22 | Diabetes annual check | |  |
| 65 | Diabetic consultation | |  |
| 97 | Insulin dosage | |  |
|  |  | |  |
| **Hospital data (HES) - ICD 10** | | |  |
| E11 | | |  |

| **Hypertension** | | |
| --- | --- | --- |
| **Readcode list** | | |
| **Medcode** | **Read code** | **Read term** |
| 1894 | G201.00 | Benign essential hypertension |
| 83473 | G203.00 | Diastolic hypertension |
| 799 | G20..00 | Essential hypertension |
| 10818 | G20z.00 | Essential hypertension NOS |
| 351 | G20..11 | High blood pressure |
| 85944 | 7Q01.00 | High cost hypertension drugs |
| 3712 | G20z.11 | Hypertension NOS |
| 15377 | G200.00 | Malignant essential hypertension |
| 3425 | 662O.00 | On treatment for hypertension |
| 101649 | 7Q01y00 | Other specified high cost hypertension drugs |
| 107704 | G20..12 | Primary hypertension |
| 105487 | G26..11 | Severe hypertension |
| 105989 | G26..00 | Severe hypertension (Nat Inst for Health Clinical Ex 2011) |
| 105316 | G25..11 | Stage 1 hypertension |
| 105371 | G25..00 | Stage 1 hypertension (NICE - Nat Ins for Hth Clin Excl 2011) |
| 105274 | G28..00 | Stage 2 hypertension (NICE - Nat Ins for Hth Clin Excl 2011) |
| 4372 | G202.00 | Systolic hypertension |
| 676 | R1y2.00 | [D]Raised blood pressure reading |
| 8574 | 662Q.00 | Borderline blood pressure |
| 4552 | 68B4.00 | Risk factors present at hypertension screen |
| 22356 | 1JD..00 | Suspected hypertension |
|  |  |  |
| **Hospital data (HES) - ICD 10** | | |
| I10-I15 | | |

**Appendix Table 2:** Drug classification

| **Action** | **Drug classification** |
| --- | --- |
| Antihypertensive | Thiazides |
|  | Loop diuretics |
|  | Potassium-sparring diuretics |
|  | Potassium-sparring diuretics & other diuretics |
|  | Osmotic diuretics |
|  | Mercurial diuretics |
|  | Carbonic Anhydrase Inhibitors |
|  | Diuretics with potassium |
|  | Beta Blockers |
|  | Vasodilators |
|  | Central Antihypertensive |
|  | Adrenergic Neurone Blockers |
|  | Alpha Blockers |
|  | ACE Inhibitors |
|  | Angiotensin II receptor blockers |
|  | Renin Inhibitors |
|  | Calcium channel blockers |
| Antidiabetic | Insulins |
|  | Sulphonylureas |
|  | Biguanides |
|  | DPP-4 inhibitors |
|  | Thiazolidinediones |
|  | Gliflozins |
|  | GLP1 agonists |
|  | Meglitinides |
|  | Acarbose |
|  | Other antidiabetic drugs |
| Lipid-lowering | Statins |
|  | Statins/Ezetimibe combination |
|  | Bile Acid Sequestrants |
|  | Fibrates |
|  | Nicotinic Acid Group |
|  | Probucol |

**Appendix Table 3:** Deficits included in the eFI

| **Disease State** | **Arthritis** |
| --- | --- |
|  | **Atrial Fibrillation** |
|  | **Chronic Kidney Disease** |
|  | **Coronary Heart Disease** |
|  | **Diabetes** |
|  | **Foot Problems** |
|  | **Fragility/Fracture** |
|  | **Heart Failure** |
|  | **Heart Valve Disease** |
|  | **Hypertension** |
|  | **Hypotension/Sincope** |
|  | **Osteoporosis** |
|  | **Parkinson’s Disease** |
|  | **Peptic Ulcer** |
|  | **Peripheral Vascular Disease** |
|  | **Respiratory Disease** |
|  | **Skin Ulcer** |
|  | **Stroke and TIA** |
|  | **Thyroid Disorder** |
|  | **Urinary System Disease** |
| **Symptoms/Signs** | **Dizziness** |
|  | **Dyspnoea** |
|  | **Falls** |
|  | **Memory and Cognitive Problems** |
|  | **Polypharmacy** |
|  | **Sleep Disturbance** |
|  | **Urinary Incontinence** |
|  | **Weight Loss and Anorexia** |
| **Disability** | **Activity Limitation** |
|  | **Hearing Loss** |
|  | **Housebound** |
|  | **Mobility and Transfer problems** |
|  | **Requirement for Care** |
|  | **Social Vulnerability** |
|  | **Vision Problems – Blindness** |
| **Abnormal laboratory value** | **Anaemia & Haematinic Deficiency** |

**Appendix Table 4:** Complete-case analysis

|  | **Men** | | | **Women** | | | **Overall** | | |
| --- | --- | --- | --- | --- | --- | --- | --- | --- | --- |
| **ADJUSTED PROPORTION** | **OR** | **95%CI** | | **OR** | **95%CI** | | **OR** | **95%CI** | |
| **Type 2 diabetes** | 1.06 | 0.83 | 1.36 | 1.01 | 0.84 | 1.22 | 1.04 | 0.90 | 1.20 |
| **Hypertension** | **1.05** | **1.04** | **1.05** | **1.06** | **1.05** | **1.06** | **1.06** | **1.05** | **1.06** |
| **Medication** |  |  |  |  |  |  |  |  |  |
| ***Anti-diabetic medication for those with diabetes*** | **0.39** | **0.22** | **0.70** | **0.38** | **0.25** | **0.58** | **0.44** | **0.32** | **0.61** |
| ***Anti-hypertensive for those with hypertension*** | **0.40** | **0.25** | **0.65** | **0.51** | **0.37** | **0.71** | **0.47** | **0.37** | **0.62** |
| ***ACE*** |  |  |  |  |  |  |  |  |  |
| ***Lipid-lowering medication*** | **0.56** | **0.42** | **0.75** | **0.74** | **0.59** | **0.92** | **0.68** | **0.57** | **0.80** |
| **ADJUSTED DIFFERENCE IN RISK FACTOR VALUES** | **Coeff.** | **95%CI** | | **Coeff.** | **95%CI** | | **Coeff.** | **95%CI** | |
| **Blood pressure** |  |  |  |  |  |  |  |  |  |
| ***Systolic blood pressure*** | -1.10 | -2.24 | 0.04 | 0.49 | -0.15 | 1.12 | 0.13 | -0.42 | 0.69 |
| ***for those with a diagnosis of hypertension at baseline*** | -1.62 | -4.28 | 1.03 | 0.28 | -1.69 | 2.26 | -0.28 | -1.85 | 1.30 |
| ***Diastolic blood pressure*** | 0.14 | -0.57 | 0.85 | **0.60** | **0.20** | **0.99** | **0.51** | **0.16** | **0.86** |
| ***for those with a diagnosis of hypertension at baseline*** | 0.30 | -1.29 | 1.90 | 0.90 | -0.29 | 2.08 | 0.77 | -0.18 | 1.72 |
| **BMI** | **-0.99** | **-1.33** | **-0.64** | **-0.38** | **-0.62** | **-0.13** | **-0.52** | **-0.72** | **-0.32** |
| ***Underweight*** | -0.02 | -0.85 | 0.82 | -0.20 | -0.44 | 0.04 | -0.20 | -0.42 | 0.03 |
| ***Normal weight*** | **-0.27** | **-0.45** | **-0.08** | **-0.14** | **-0.25** | **-0.04** | **-0.18** | **-0.27** | **-0.08** |
| ***Overweight*** | -0.12 | -0.28 | 0.04 | 0.01 | -0.10 | 0.13 | -0.03 | -0.13 | 0.06 |
| ***Obese*** | -0.36 | -1.03 | 0.31 | -0.20 | -0.63 | 0.23 | -0.23 | -0.60 | 0.13 |
